# Supplementary material for: A selectivity filter mutation provides insights into gating regulation of a K+ channel
Source: Commun Biol. 2022 Apr 11;5:345. doi: 10.1038/s42003-022-03303-1 (PMC9001731; doi:10.1038/s42003-022-03303-1)
Supplement: Supplementary file 2 — Supplementary Information [file 42003_2022_3303_MOESM2_ESM.pdf]

SUPPLEMENTARY INFORMATION

A selectivity filter mutation provides insights into gating regulation of a K^+ channel

Authors: Friesacher et al.

This PDF includes:

Supplementary Figures 1-3

Supplementary Tables 1, 2

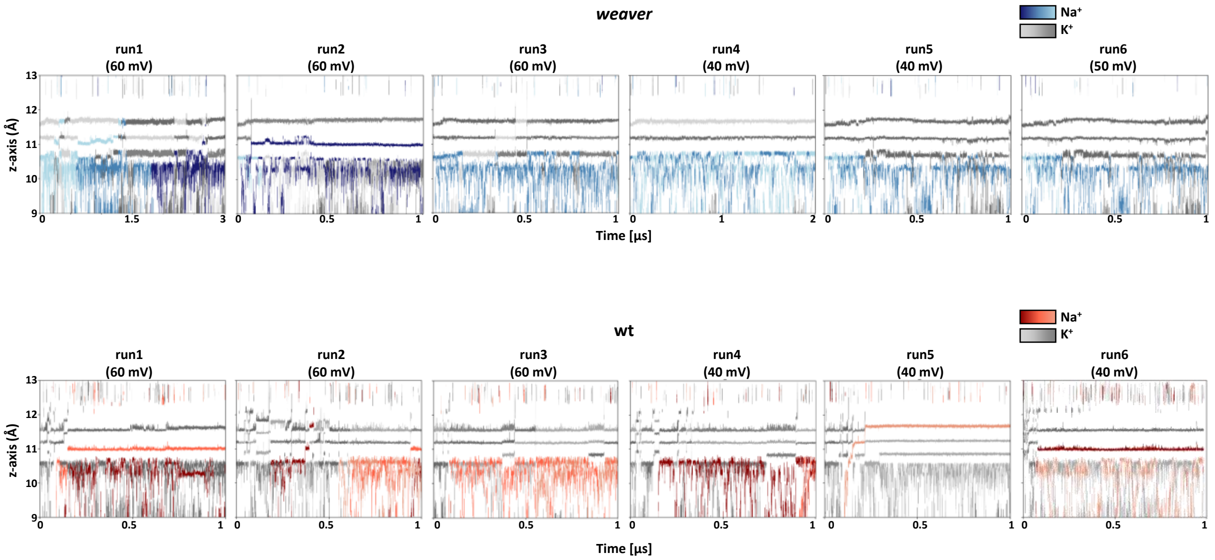

Supplementary Figure 1. Ion flux through the mGIRK2$_{wv}$ (top) and mGIRK2$_{wt}$ (bottom) SF over simulation time. Each line displays the position of one ion or H_2O molecule on the z-axis over simulation time. K^+ is shown in grey, Na^+ in shades of blue and orange for mGIRK2$_{wv}$ and mGIRK2$_{wt}$, respectively.

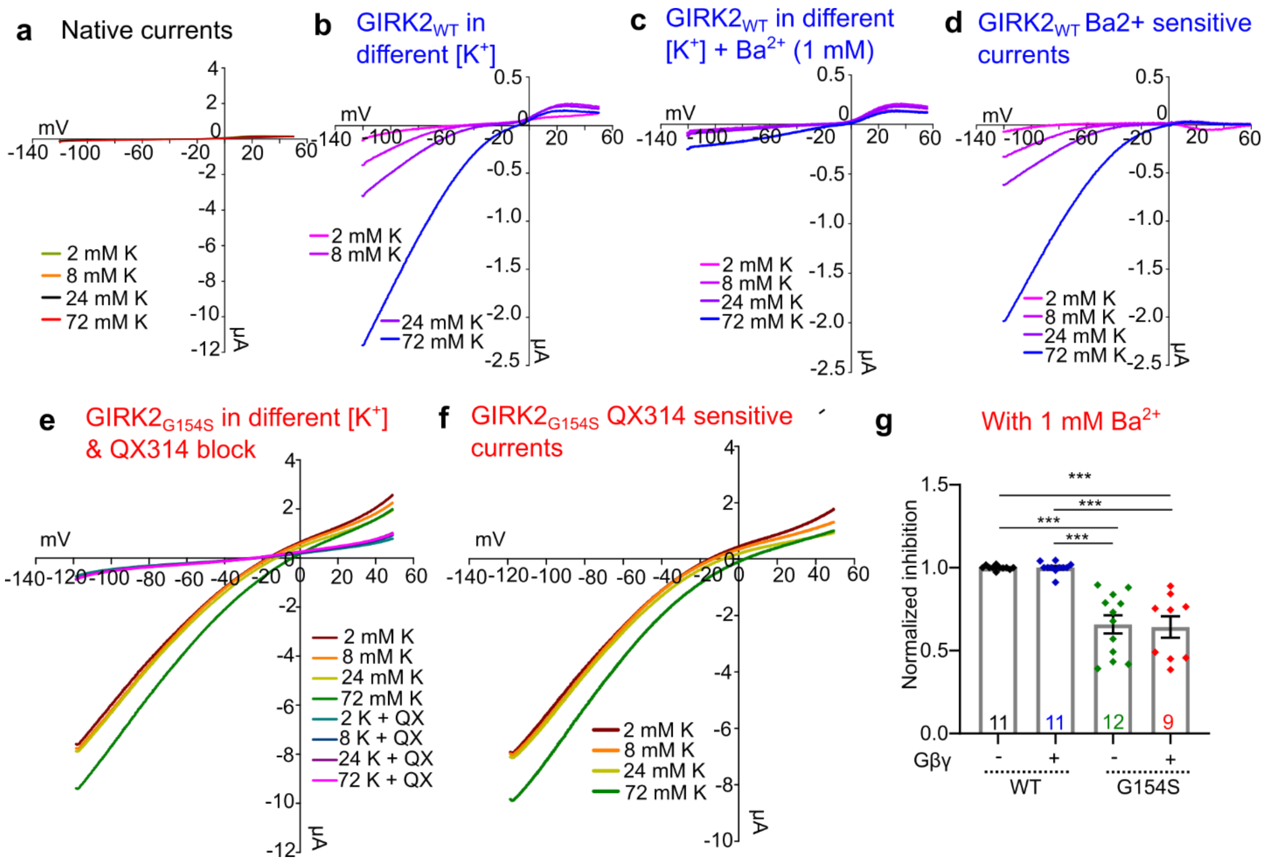

Supplementary Figure 2. The effect of Ba^{2+} and QX314 on naïve as well as hGIRK2$_{wt}$ and hGIRK2$_{G154S}$-expressing oocytes. a Average currents from naïve oocytes obtained in four external K+ concentrations (n=5, one experiment). **b-d** Exemplary I-V relationships of hGIRK2$_{wt}$ obtained in four external K+ concentrations. I-V curves obtained in the presence of 1 mM Ba^{2+} (c) were subtracted from I-V curves without the blocker (b), yielding net GIRK I-V curves (d). **e** Exemplary I-V relationships of hGIRK2$_{G154S}$ obtained in four external K+ concentrations with and without 400 μM QX314. **f** I-V curves of QX sensitive currents in four external K+ concentrations. **g** Normalized data of % inhibition by 1 mM Ba^{2+} on hGIRK2$_{wt}$ vs hGIRK2$_{G154S}$ with and without Gβγ (N=2 independent experiments). Number of oocytes are shown within the bars. One way ANOVA followed by Kruskal-Wallis test. ***$p<0.001$

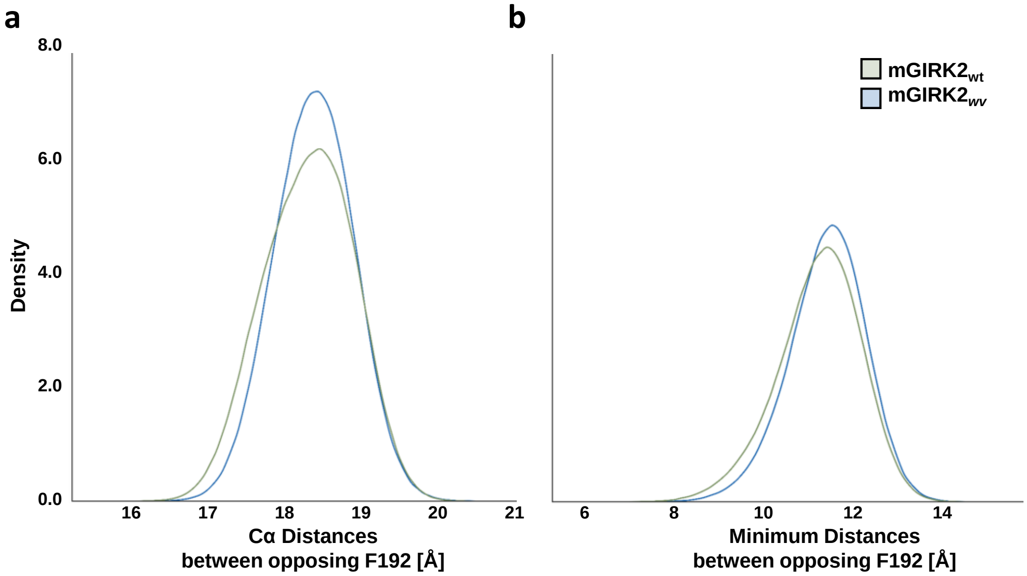

Supplementary Figure 3. Pore dimensions at the HBC gate over 6 µs mGIRK2$_{wt}$ and 9 µs mGIRK2$_{wv}$ MD simulation. **a** Distribution of the distances between the Cα of opposing F192. **b** Distribution of minimum distances between opposing F192.

Supplementary Table 1. List of simulated systems and observed number of full ion permeation events.

		Simulation Time (μs)	*E*lectric Field (mV nm^{-1})	**K^+** *PE*†	**Na^+** *PE*†
**mGIRK2$_{wt}$**	run1	1	60	4	0
	run2	1	60	18	1
	run3	1	60	4	0
	run4	1	40	7	0
	run5	1	40	3	0
	run6	1	40	4	0
	**Total**	**6**		**40**	**1**
**mGIRK2$_{wv}$**	run1	3	60	10	4
	run2	1	60	0	0
	run3	1	60	3	0
	run4	2	40	0	0
	run5	1	40	0	0
	run6	1	50	0	0
	**Total**	**9**		**13**	**4**

† full permeation events (PE) through the SF

Supplementary Table 2. List of chemicals used for electrophysiological experiments.

**Bacterial strains**		
DH5α	New England Biolabs	C2987I
**Chemicals, Peptides, and Recombinant Proteins**		
QX314	Alomone labs	#Q-150
Barium Chloride ($BaCl_2$)	Merck	1719.0500
Potassium chloride (KCl)	Merck	1.04936.1000
Sodium chloride (NaCl)	Merck	1.06404.1000
Magnesium chloride ($MgCl_2$.6H20)	Merck	1.05833.1000
Calcium Chloride ($CaCl_2$)	Sigma-Aldrich	C1016
HEPES	Biological industries	41-122-100
Sodium Hydroxide (NaOH)	Merck	1.06498.1000
Potassium Hydroxide (KOH)	Merck	1.05033.1000
Methanesulfonic acid	Sigma-Aldrich	471356
Collagenase-Type 1A	Sigma-Aldrich	C9891-1G
Na-Pyruvate	Sigma-Aldrich	P2256
Gentamycin Sulfate Solution, 50mg/ml	Biological industries	03-035-1b
**Critical Commercial Assays**		
Miniprep Kit	Promega	A1460
PWO master PCR	Roche	03789403001
Nhe1	New England Biolabs	R3131S
SalI	New England Biolabs	R3138S
**Experimental Models: Cell Lines/ primary cell culture**		
Xenopus laevis oocytes	Xenopus-1	http://www.xenbase.org/community/org.do?orgId=1365&method=Display
**Oligonucleotides**		
**DNA primers for mutagenesis**		
G154S<br>**Forward primer:**<br>CAGAGACAACCATCAGCTACGGCTACAG<br>**Reverse primer:**<br>CTGTAGCCGTAGCTGATGGTTGTCTCTG	This study	
**Recombinant DNA**		
Human GIRK2	Blavatnik center, TAU	NM_0O2240
Human GIRK2$_{G154S}$	This study	
Bovine Gβ_1	Dascal Lab	NM_175777.3
Bovine Gγ_2	Dascal Lab	NM_174072.4
**Software and Algorithms**		
Prism 8	GraphPad	https://www.graphpad.com/scientific-software/prism/
SigmaPlot 11 or 13	Systat Software, Inc.	https://systatsoftware.com/products/sigmaplot/sigmaplot-version-13/
pClamp 10.5	Molecular Devices	https://www.moleculardevices.com
**OTHER**		
Geneclamp 500	Molecular Devices	https://www.moleculardevices.com
